# Multi-Trait Characterization and Selection of Elite Wild Kiwifruit (*Actinidia* spp.) Germplasm from the Dabie Mountains of Hubei, China

**DOI:** 10.3390/plants15192894

**Published:** 2026-09-22

**Authors:** Wei Huang, Zhiyuan Zhao, Hong Zhang, Jingwen Yang, Jinghua Zhai, Hongjin Dong, Zhiwei Chen, Xiuli Li, Wei Tu, Xiaomei Ji

**Affiliations:** 1 Wuhan Academy of Agricultural Sciences, No. 173 Baishazhou Avenue, Hongshan District, Wuhan 430065, China; huangwei@wuhanagri.com (W.H.); zhaozhiyuan@wuhanagri.com (Z.Z.); zhanghong@wuhanagri.com (H.Z.); yangjingwen@wuhanagri.com (J.Y.); zhaijinghua0308@126.com (J.Z.); thincker@163.com (Z.C.); lixiuli@wuhanagri.com (X.L.); 2 Hubei Key Laboratory of Economic Forest Germplasm Improvement and Resources Comprehensive Utilization, Hubei Collaborative Innovation Center for the Characteristic Resources Exploitation of Dabie Mountains, Huanggang Normal University, Huanggang 438000, China; hongjind@hgnu.edu.cn

**Keywords:** Dabie Mountains, wild kiwifruit, germplasm resources, fruit quality, bacterial canker resistance, heat tolerance

## Abstract

Wild kiwifruit germplasm can broaden breeding pools through complementary fruit-quality and stress-response traits. The Hubei Dabie Mountains contain heterogeneous habitats, but coordinated evaluation of their wild *Actinidia* resources remains limited. Taxonomy and ploidy were determined for 117 accessions from eight sites spanning 170–1730 m. Nine descriptive fruit traits and detached-leaf responses to bacterial canker and heat were assessed in 41 fruiting accessions. Bacterial canker response was assessed using *Pseudomonas syringae* pv. *actinidiae* (Psa), and heat response using maximum photosystem II quantum efficiency (F_v_/F_m_) after 47 °C treatment. Seven fruit-quality traits were measured in 27 accessions with normally ripened fruit. Three taxa were identified: *A. chinensis*, *A. polygama*, and *A. chinensis* var. *deliciosa*. Diploid, tetraploid, and hexaploid cytotypes were detected. Shannon–Wiener indices for descriptive traits ranged from 0.26 to 1.64. Twenty-three accessions were classified as highly or moderately resistant to Psa. Heat screening classified 23 accessions as heat tolerant, 16 as moderately heat tolerant, and two as heat sensitive. Fruit-quality coefficients of variation ranged from 12.92% to 120.41%. Fruit-quality ranking followed by Psa screening identified MC-021, MC-026, YS-031, YS-037, and YS-038 as candidate elite germplasm. These accessions are priorities for conservation and further breeding evaluation.

## 1. Introduction

Kiwifruit was domesticated from wild plants during the twentieth century. Its nutritional value is associated with vitamin C, dietary fiber, and minerals [1,2]. China is a center of origin and the principal center of diversity for *Actinidia*, with extensive wild germplasm [2]. In 2024, kiwifruit was cultivated on 178,200 ha in China, with production totaling 2.1535 million t [3]. These values represented 65.63% of global cultivated area and 48.75% of global production [3]. However, mean yield was approximately 12.08 t ha^−1^, one-quarter of New Zealand’s yield and below the global average [3]. This yield difference cannot be attributed to germplasm alone. Productivity is influenced by cultivar background, disease and heat responses, and cultivation practices [4,5,6,7]. Broadening the breeding gene pool could provide additional combinations of favorable fruit traits and stress responses [2,4,5,6].

Wild germplasm provides genetic variation for kiwifruit improvement. Contrasting mountain environments expose wild populations to different selection pressures. Genetic variation within *Actinidia* is also shaped by interspecific hybridization, gene flow, and polyploidization [2,8]. The taxonomy of Actinidiaceae in China was described by Li et al. [9]. Huang documented 54 species and 21 varieties of *Actinidia*, including 73 taxa occurring in China [10]. A genus-level super-pangenome was constructed from 14 haplotype genomes representing seven diploid *Actinidia* materials [11]. Structural variants associated with fruit-size and disease-resistance differences were identified, providing genomic context for phenotypic evaluation [11]. Broad variation in sugars, acids, dry matter, soluble solids, pigments, and ascorbic acid has been reported in wild *Actinidia eriantha* [12,13]. Wild *Actinidia* accessions with limited symptoms after Psa inoculation have also been identified [4]. Favorable physiological responses to heat treatment have been recorded in selected materials [5]. Regional surveys and coordinated evaluation can therefore support germplasm conservation, parent selection, rootstock development, and cultivar breeding [2,4,5,12,13,14,15].

Hubei Province is a center of kiwifruit origin, with production concentrated in the Wuling, Mufu, Qinba, and Dabie mountain regions [16]. Wild kiwifruit resources have been evaluated in several of these regions. In Tongshan County, 152 wild *Actinidia* accessions were recorded across seven taxa, including diploid and tetraploid materials [17]. Extensive variation in fruit traits was observed in this collection [17]. Wild resources from the Wudang Mountains were collected, conserved ex situ, and evaluated, resulting in the cultivar ‘Wudang 1’ [18]. These studies demonstrate the breeding relevance of Hubei mountain germplasm. Comparable information remains limited for the Hubei Dabie Mountains. Regional taxonomic composition, ploidy structure, fruit quality, and stress responses have not been evaluated together. Their coordinated assessment could guide conservation and identify accessions for further breeding evaluation.

The Hubei Dabie Mountains span 29°45′–31°48′ N and 114°00′–116°07′ E. The landscape comprises mid-elevation mountains, low mountains, hills, and plains. Tiantangzhai reaches 1729 m [19]. Mean annual temperature is 14–17 °C, annual precipitation is 1100–1400 mm, and the frost-free period lasts 230–270 d [19]. Approximately 70–75% of annual rainfall occurs from April to July [19]. Diverse habitats are provided by the monsoon climate and topographic variation. Three objectives were defined for this study. First, taxonomic and cytotype composition was documented for 117 accessions from eight population sites. Second, descriptive fruit traits, Psa response, and post-treatment F_v_/F_m_ were evaluated in 41 fruiting accessions. Fruit-quality assessment was restricted to 27 accessions with normally ripened fruit. Third, candidates were selected through fruit-quality ranking followed by Psa screening, with heat-response classes reported for further characterization. This design links the surveyed collection to defined evaluation subsets and identifies candidates for subsequent validation.

## 2. Results

### 2.1. Survey and Sampling

Eight wild kiwifruit population sites were surveyed in Huanggang City. The sites were located in Macheng City and Yingshan, Tuanfeng, and Luotian counties. They spanned 30°52′7″–31°30′21″ N, 114°47′43″–115°48′8″ E, and elevations of 170–1730 m. In total, 117 accessions were recorded, and 41 met the fruit-sampling criterion for subsequent assessment (Tables S1 and S5). Three taxa were identified morphologically: *Actinidia chinensis* Planch., *A. polygama* (Siebold & Zucc.) Maxim., and *A. chinensis* var. *deliciosa* A.Chev. These represented two species and one variety. External variation in representative mature fruits is shown in Figure 1A. External views and transverse and longitudinal fruit sections of MC-005, YS-031, and YS-040 are shown in Figure 1B. Paired leaf surfaces and one-year-old shoots of YS-016, YS-020, and YS-021 are shown in Figure 1C.

### 2.2. Ploidy Identification

Flow cytometry detected diploid, tetraploid, and hexaploid cytotypes among the 117 wild kiwifruit accessions. Of these, 109 were diploid (93.16%), three were tetraploid (2.56%), and five were hexaploid (4.27%) (Table S2). All 106 *A. chinensis* accessions were diploid. Among the eight *A. chinensis* var. *deliciosa* accessions, three were tetraploid and five were hexaploid. All three *A. polygama* accessions were diploid.

### 2.3. Diversity of Descriptive Fruit Traits

Nine descriptive fruit traits were evaluated in 41 wild kiwifruit accessions (Table 1). Sepals were persistent in 48.78% of accessions and absent in 51.22%. Fruit shoulders were predominantly square or circular. Fruit apices were concave (43.90%), flat (29.27%), or convex (26.83%). Shallow and deep blunt protrusions were the predominant fruit-beak forms. Most fruits lacked pubescence. Short-oval fruits were most frequent (34.15%), followed by short-cylindrical fruits (26.83%). Trapezoidal, globose, obovate, ellipsoid, and long-ellipsoid fruits occurred less frequently. Circular fruit cross-sections accounted for 85.37% of accessions. Core cross-sections were mainly irregular, followed by elliptic and circular forms. Flesh color was predominantly green or yellow-green, whereas yellow flesh was less frequent.

Figure 2 summarizes the scoring categories and phenotypic diversity of the nine descriptive fruit traits. Shannon–Wiener index (H′) values ranged from 0.26 to 1.64, with a mean of 0.91. Fruit shape had the highest H′ value (1.64). Fruit pubescence, fruit cross-sectional shape, and sepal persistence had H′ values below 0.70. These traits therefore showed relatively concentrated phenotypic distributions within the collection.

### 2.4. Diversity of Quantitative Fruit Traits

After harvest, fruits were stored at room temperature until they reached the soft-ripe stage. Fruits from 14 of 41 accessions developed severe dehydration or soft rot and were excluded from further analysis. Fruit quality was therefore evaluated in the remaining 27 accessions. Traits included fruit weight, longitudinal diameter, transverse diameter, soluble solids content, titratable acidity, dry matter content, and vitamin C content. Longitudinal diameter ranged from 2.88 to 5.85 cm, with a mean of 3.80 cm. Transverse diameter ranged from 2.59 to 4.46 cm, with a mean of 3.39 cm. The fruit shape index ranged from 0.90 to 1.52, with a mean of 1.12. Most fruits were nearly round or short cylindrical. Fruit weight ranged from 10.72 to 60.01 g, with a mean of 26.33 g. Seven accessions produced fruits weighing more than 30 g. Soluble solids content ranged from 9.17% to 18.87%, with a mean of 13.06%. Titratable acidity ranged from 0.36% to 1.62%, with a mean of 0.83%. Dry matter content ranged from 13.64% to 28.86%, with a mean of 18.41%. Vitamin C content ranged from 3.22 to 149.77 mg 100 g^−1^ fresh weight (FW), with a mean of 26.03 mg 100 g^−1^ FW. Most accessions had low or intermediate vitamin C contents.

Variation in seven fruit-quality traits among the 27 accessions is summarized in Table 2. Coefficients of variation ranged from 12.92% to 120.41%, with a mean of 38.97%. The highest coefficient was recorded for vitamin C content (120.41%). Lower coefficients were recorded for dry matter content (17.61%) and transverse diameter (12.92%). H′ values ranged from 1.50 to 1.88, with a mean of 1.78. Substantial phenotypic variation was therefore observed within the normally ripened subset.

### 2.5. Bacterial Canker Resistance of Wild Kiwifruit Resources from the Dabie Mountains

Psa response was evaluated in the same 41 accessions used for descriptive fruit assessment. This common panel was used to support comparisons across traits, although fruit was not required for detached-leaf inoculation. Lesion areas ranged from 0.02 to 39.38 cm^2^ at 14 d after inoculation. Two accessions were classified as highly resistant and 21 as moderately resistant. Seven were classified as susceptible, three as moderately susceptible, and eight as highly susceptible (Figure 3). These frequencies describe the evaluated panel.

### 2.6. Heat Tolerance of Wild Kiwifruit Resources from the Dabie Mountains

Heat response was evaluated in the same 41-accession panel using detached leaves exposed to a 47 °C water bath. Maximum quantum efficiency of photosystem II (F_v_/F_m_) was measured after treatment (Figure 4). Post-treatment F_v_/F_m_ ranged from 0.74 to 0.85, with a mean of 0.82. The coefficient of variation was 2.43%, and H′ was 1.80. Three heat-response classes were defined by optimal partitioning of ordered data. Accessions were classified as heat tolerant at F_v_/F_m_ ≥ 0.82 and heat sensitive at F_v_/F_m_ ≤ 0.78. Values between these thresholds were classified as moderately heat tolerant. The three classes contained 23 heat-tolerant, 16 moderately heat-tolerant, and two heat-sensitive accessions. These represented 56.10%, 39.02%, and 4.88% of the evaluated panel, respectively.

### 2.7. Cluster Analysis

Hierarchical clustering was performed for 27 accessions using nine z-standardized variables. These comprised the Psa disease index, post-treatment F_v_/F_m_, and seven fruit-quality traits (Figure 5). Five groups were distinguished at a Euclidean distance of 3.82. Group I contained 17 accessions with relatively large longitudinal and transverse diameters and high post-treatment F_v_/F_m_. Group II contained three accessions with lower Psa disease indices and higher dry matter and soluble solids contents. Group III contained five accessions with relatively high fruit weight. Group IV contained only YS-002, with a low Psa disease index and comparatively low post-treatment F_v_/F_m_ and vitamin C content. Group V contained only MC-004, with high vitamin C content and a high Psa disease index. Fruit weight and the two diameter variables remained correlated after standardization. Fruit size could therefore exert disproportionate influence on the distances. The clusters were interpreted as an exploratory description of trait combinations and were excluded from ranking and candidate selection.

### 2.8. Principal Component Analysis and Selection of Candidate Germplasm

Principal component analysis was applied to seven fruit-quality traits from 27 wild kiwifruit accessions. Eigenvalues and variance contributions are summarized in Table 3, while trait loadings are presented in Table 4. Three components with eigenvalues above one were retained. Their eigenvalues were 2.880, 1.786, and 1.157, and together they explained 83.172% of total variance. The first component explained 41.136% of variance and loaded strongly on fruit weight and longitudinal and transverse diameters. It primarily represented fruit size. The second component explained 25.513% of variance and loaded strongly on soluble solids and dry matter contents. It primarily represented compositional quality. The third component explained 16.523% of variance and loaded strongly on vitamin C content and titratable acidity. It represented variation associated with fruit acidity and nutritional quality.

Three principal component score functions were derived from the coefficients of the standardized variables:

F_1_ = −0.052X_1_ + 0.049X_2_ − 0.049X_3_ + 0.102X_4_ + 0.347X_5_ + 0.317X_6_ + 0.340X_7_

F_2_ = 0.497X_1_ + 0.534_2_ − 0.118X_3_ + 0.103X_4_ − 0.009X_5_ + 0.050X_6_ + 0.049X_7_

F_3_ = 0.026X_1_ − 0.038X_2_ + 0.639X_3_ + 0.629X_4_ + 0.032X_5_ − 0.138X_6_ + 0.169X_7_

Here, X_1_–X_7_ represent dry matter content, soluble solids content, titratable acidity, vitamin C content, fruit weight, longitudinal diameter, and transverse diameter, respectively. Each component’s variance contribution was divided by the cumulative contribution of the first three components. The resulting ratios were used as weights to calculate the comprehensive fruit-quality score (F):

F = 0.4946F_1_ + 0.3067F_2_ + 0.1987F_3_

Component scores, comprehensive fruit-quality scores, and rankings are presented in Table 5. Higher values indicate higher scores under the specified variance-weighted model. Seven accessions exceeded 0.5: YS-038, MC-004, MC-021, YS-037, MC-026, YS-033, and YS-031. Psa response was then applied as a mandatory filter. Highly susceptible MC-004 and susceptible YS-033 were excluded. MC-021, MC-026, YS-031, YS-037, and YS-038 were retained because they were classified as highly or moderately resistant. Heat-response class was reported for characterization and was not used as an exclusion criterion. Accordingly, moderately heat-tolerant YS-031 and YS-038 were retained.

## 3. Discussion

The surveyed collection provides a basis for relating regional germplasm diversity to candidate selection. Conservation planning is supported by taxonomic and cytotype records for 117 accessions. Descriptive fruit traits, Psa response, and heat response can be compared directly within the common panel of 41 accessions. Fruit-quality ranking was restricted to the 27 accessions that ripened normally. The progressively smaller subsets define the scope of inference at each stage. The five selected accessions should therefore be prioritized for validation before their suitability as breeding parents is established.

Regional differences in cytotype composition were evident. All 106 *A. chinensis* accessions in the surveyed collection were diploid, whereas 125 of 133 *A. chinensis* accessions from Tongshan County were tetraploid [17]. The present taxon–ploidy associations were consistent with a wider survey of 3338 individuals in the *A. chinensis* complex [20]. Diploids were associated with *A. chinensis*, hexaploids with *A. chinensis* var. *deliciosa*, and tetraploids with both taxa [20]. However, few *A. chinensis* var. *deliciosa* and *A. polygama* accessions were represented in the present collection. Regional differences in cytotype origin or adaptation cannot be resolved from these observations. The records can nevertheless inform conservation and the choice of materials for ploidy-compatible crosses.

Variation in fruit traits provides opportunities to select materials for different quality targets. Fruit shape was more diverse than traits such as fruit pubescence within the 41-accession panel. Substantial variation in composition was also observed among the 27 normally ripened accessions, particularly for vitamin C content. Similar variation in fruit morphology and quality was reported in Tongshan County [17]. Differences in major compositional traits were also recorded among 145 wild *A. eriantha* genotypes from Jiangxi [12]. These findings support evaluation of wild germplasm for fruit size, flavor, processing, and nutritional traits. However, measurements in the present study were obtained from different habitats during one harvest year. Genetic and environmental contributions to the observed phenotypic differences cannot be separated without evaluation under common conditions.

The detached-leaf responses identify accessions for further resistance testing, but neither resistance mechanisms nor field stability can be inferred from lesion phenotypes alone. In a three-year detached-shoot study, 104 wild *Actinidia* genotypes were evaluated, and variation was observed both among and within species [4]. A tetraploid *A. arguta* genotype was classified as highly resistant in that study [4]. Tetraploid and hexaploid cytotypes have also been documented in a natural *A. arguta* population [21]. These findings support the inclusion of *A. arguta* in future resistance-breeding studies, with ploidy considered when selecting parents. The reported resistance belongs to the tested genotype and assay conditions and should not be generalized to all *A. arguta* cytotypes. *A. arguta* could be included as resistant reference material in future assays and evaluated as a potential donor for interspecific breeding. Cross compatibility and resistance inheritance would require assessment before its use as a parent. Direct resistance comparisons require equivalent inoculation methods, because detached-leaf and detached-shoot responses are not interchangeable [22]. Differences among studies may also reflect bacterial strain, environment, or classification criteria. Genotype-associated bacteria isolated from a resistant kiwifruit cultivar suppressed canker symptoms in inoculated shoots [23]. In *A. chinensis* var. *chinensis*, resistance was enhanced by overexpression of *AcREM14* and *AcC3H1*, with increased salicylic-acid-associated defense responses [24]. Neither microbiome composition nor these regulatory mechanisms were measured in the present study. Their contributions could be examined in accessions with contrasting Psa responses, alongside detached-shoot and field validation.

Post-treatment F_v_/F_m_ can support preliminary screening of heat responses under standardized conditions. Maximum PSII quantum efficiency is assessed using F_v_/F_m_ in dark-adapted leaves, allowing stress-related changes in photochemistry to be detected [25]. A 47 °C treatment for 40 min was established for detached grape leaves by Xu et al. [26]. The same conditions were applied to 247 Chinese wild grape accessions over three years, with relatively stable accession rankings [27]. Higher F_v_/F_m_ and more stable photosynthesis were also observed in a heat-tolerant *A. chinensis* mutant than in its wild type [28]. These studies support fluorescence-based assessment, although treatment sensitivity and thresholds require validation in the target material. Most accessions in the present panel retained relatively high post-treatment F_v_/F_m_. However, the heat-response classes were defined from this panel and should not be treated as universal tolerance thresholds. Without untreated controls, treatment-induced declines cannot be quantified. Recovery measurements are also required to distinguish transient responses from sustained injury.

Clustering and fruit-quality ranking served different purposes. Correlated fruit weight and diameter variables gave fruit size greater collective representation in clustering than individually represented traits. Standardization removed differences in scale but did not eliminate this redundancy. Cluster membership was therefore excluded from selection decisions. PCA was restricted to fruit-quality traits, so the ranking did not incorporate Psa response or F_v_/F_m_. Nevertheless, fruit size also contributed strongly to PC1 and therefore influenced the comprehensive score. Variance-based weights describe variation in this dataset rather than independently established breeding preferences. A higher score should consequently be interpreted within this model, particularly because increasing titratable acidity is not necessarily desirable for every quality target. Seven accessions with scores above 0.5 were shortlisted, and the Psa filter retained five. MC-004 and YS-033 were excluded because of their susceptibility classes. Heat response was reported separately, allowing moderately heat-tolerant YS-031 and YS-038 to remain candidates. The resulting selections require validation against defined breeding targets. Accessions with exceptional individual traits, including MC-004, should also be conserved.

Further evaluation should address the limitations imposed by field sampling and assay design. Only 41 surveyed accessions supplied sufficient fruit during the visits, and only 27 yielded normally ripened fruit for quality assessment. Exclusion of nonfruiting accessions and those with abnormal ripening may have introduced selection bias. Habitat and year effects also prevent the present measurements from establishing trait stability. Ex situ conservation and propagation should be followed by common-garden assessment over multiple years. Psa resistance should be tested with additional strains, detached-shoot inoculation, and field observations. Heat responses should be assessed using untreated controls, temperature gradients, recovery measurements, and complementary physiological traits. Accessions with reproducible performance could then be considered for crossing, rootstock evaluation, or regional adaptation trials.

## 4. Materials and Methods

### 4.1. Field Survey and Material Collection

Wild kiwifruit germplasm was surveyed in mid-September and early October 2024. Four administrative areas in the Hubei Dabie Mountains were covered: Macheng, Yingshan, Tuanfeng, and Luotian. Collection sites are listed in Table S1. Published distribution records were reviewed before fieldwork to guide the survey [29]. Assistance was provided by researchers from Huanggang institutions and local guides familiar with the terrain and wild resources. Latitude, longitude, and elevation were recorded using a smartphone-based positioning system. Each accession represented one independent wild vine sampled in situ, rather than a clone or a pooled sample from a site. Taxonomic identification was based on morphological characteristics following Huang [10]. Species were not selected in advance; the three recorded taxa were identified among the 117 vines collected from the eight sites. Six semilignified shoots, each approximately 20 cm long, were collected per accession for ploidy analysis. Fruit was collected when available. At least five fruits at a suitable developmental stage were required per accession for subsequent assessment. Thirty mature functional leaves were collected per fruiting accession for Psa and heat-response assays. Only 41 accessions supplied at least five fruits at a suitable developmental stage during the two visits. The 41-accession subset was defined by fruit availability and developmental stage, rather than measured trait performance. Descriptive fruit traits, Psa response, and heat response were evaluated in this common panel to support comparisons across traits. Although fruit was not required for either leaf assay, both assays were restricted to these 41 accessions. During postharvest ripening, fruit from 14 accessions developed soft rot or severe shriveling or dehydration before reaching a comparable edible-ripe stage. Fruit-quality analysis was restricted to the remaining 27 accessions. Participation in each analysis is listed in Table S5.

### 4.2. Experimental Methods

#### 4.2.1. Chromosome Ploidy Identification

The diploid *A. chinensis* cultivar ‘Hongyang’ (2n = 2x = 58) served as the reference standard. Ploidy was determined in young leaves from hydroponically grown shoots. A CyFlow™ Ploidy Analyser flow cytometer was used (Sysmex Partec GmbH, Görlitz, Germany). A young leaf segment approximately the size of a fingernail was placed in a clean Petri dish. CyStain UV Precise P lysis buffer was added at 200 µL (Sysmex Partec; catalogue no. 05-5002). Polyvinylpyrrolidone was also added at 2 mg (PVP; Biosharp (Biosharp Technology Co., Ltd., Beijing, China) catalogue no. BS912-100 g). The leaf was rapidly chopped with a blade, followed by the addition of 1.8 mL ultrapure water. After mixing, the suspension was passed through a 400-mesh filter and collected in a centrifuge tube. Flow-cytometric histograms were recorded and exported automatically, and peak plots were retained.

#### 4.2.2. Fruit Trait Description and Quality Determination

Nine descriptive traits were recorded in postharvest fruit according to Descriptors for Kiwifruit Germplasm Resources [30]. These comprised sepal persistence, fruit shoulder, fruit apex, fruit beak, pubescence, fruit shape, fruit cross-section, core cross-section, and flesh color. Category scores were used to calculate frequencies and phenotypic diversity indices. The scoring criteria are provided in Table S3.

Fruit weight was measured using an electronic balance. Longitudinal and transverse diameters were measured using an MNT-150 digital vernier caliper (Shanghai Meinaite Industrial Co., Ltd., Shanghai, China). Dry matter content was determined by drying samples at 65 °C to constant weight. For each replicate, 300 µL of kiwifruit juice was analyzed using a PAL-BX/ACID 8 handheld refractometer (Atago, Tokyo, Japan). Soluble solids content was expressed as a percentage. The original juice was diluted 50-fold. Titratable acidity was measured in 600 µL of diluted juice using the same instrument and was expressed as a percentage. Vitamin C content was measured using an ascorbic acid assay kit. The kit was supplied by Beijing Boxbio Science & Technology Co., Ltd., Beijing, China (catalogue no. AKV1005U). Results were expressed as mg 100 g^−1^ FW. Three fruits were measured per accession, and their mean was reported.

#### 4.2.3. Detached-Leaf Inoculation for Bacterial Canker Resistance Evaluation

Kiwifruit bacterial canker is caused by *Pseudomonas syringae* pv. *actinidiae* (Psa). Strain PSAM228 was maintained by the Fruit Tree Disease Pathogen Biology and Integrated Management Research Group at Northwest A&F University. Professor Lili Huang supplied the strain. Detached-leaf inoculation was adapted from Deng et al. [31] and Collina et al. [32]. Three healthy, disease-free functional leaves of similar size were selected from each accession. Leaves were immersed in 70% ethanol for 1 min and rinsed three times with sterile water. They were then placed on sterile filter paper in trays. A sterile 1 mL syringe was used to puncture each leaf at approximately 1 cm intervals. Each leaf received 100 punctures. An activated PSAM228 suspension was adjusted to OD_600_ = 0.0001, corresponding to approximately 10^5^ CFU mL^−1^. Both leaf surfaces were sprayed three times to distribute the suspension evenly. Leaves were air-dried in a laminar-flow cabinet, and the trays were sealed with plastic film. Samples were incubated at 16–18 °C under a 16 h light/8 h dark photoperiod. Light intensity was 5000 lx, and relative humidity was 90%. Leaves were photographed 14 d after inoculation. Lesion area was quantified using ImageJ2, and the scoring criteria are provided in Table S4.

#### 4.2.4. Heat-Tolerance Evaluation of Detached Leaves Under High-Temperature Stress

Detached-leaf heat treatment followed Liu et al. [27]. Three healthy leaves from each accession were immersed in a 47 °C water bath for 40 min. Surface water was removed after treatment. Each leaf was cut into 1 cm × 1 cm sections and dark-adapted at room temperature for 20 min. Maximum PSII quantum efficiency (F_v_/F_m_) was measured using an IMAGING-PAM MAXI chlorophyll fluorescence imaging system (Heinz Walz GmbH, Effeltrich, Germany).

#### 4.2.5. Data Processing and Analysis

Phenotypic diversity was quantified using the Shannon–Wiener index [33,34]:

H′ = −∑P_i_ ln P_i_

where P_i_ denotes the proportion of samples assigned to the ith category of a trait. The disease index was calculated as follows:

Disease index = [Σ (number of diseased leaves × disease-grade value)/(total number of leaves assessed × highest disease-grade value)] × 100

Resistance classes were assigned using the disease index, with the assay adapted from Collina et al. [32]. Highly resistant accessions had indices ≤ 10, and moderately resistant accessions had indices >10 to ≤30. Susceptible accessions had indices >30 to ≤50. Moderately susceptible accessions had indices >50 to ≤70, and highly susceptible accessions had indices > 70. Data were organized using WPS Excel. Analysis of variance and principal component analysis were performed using IBM SPSS Statistics 27.0. All variables were z-standardized before PCA and clustering. Seven fruit-quality traits were included in PCA. Three components were retained, and their scores were weighted by normalized variance contributions to calculate the comprehensive fruit-quality score. Hierarchical clustering was performed in OriginPro 2026 (version 10.3.0.180) using nine variables, Euclidean distance, and average linkage. Correlated size variables remained redundant after standardization. Clustering was therefore restricted to exploratory interpretation and was excluded from rankings and candidate selection. Accessions with comprehensive fruit-quality scores above 0.5 were shortlisted. Only shortlisted accessions classified as highly or moderately resistant to Psa were retained as candidates. Heat-response class was reported for characterization and was not used as an exclusion criterion. Figures were prepared using Origin and Matplotlib (Version3.10.9).

## 5. Conclusions

A total of 117 wild kiwifruit accessions were documented from eight sites in the Hubei Dabie Mountains. The collection comprised *A. chinensis*, *A. polygama*, and *A. chinensis* var. *deliciosa*, with diploid, tetraploid, and hexaploid cytotypes. Within the 41-accession panel, Shannon–Wiener indices for nine descriptive fruit traits ranged from 0.26 to 1.64. Twenty-three accessions were classified as highly or moderately resistant to Psa. Heat screening classified 23 accessions as heat tolerant, 16 as moderately heat tolerant, and two as heat sensitive. Among 27 accessions with normally ripened fruit, fruit-quality coefficients of variation ranged from 12.92% to 120.41%, with the greatest relative variation in vitamin C content. Seven accessions were shortlisted by fruit-quality PCA, after which highly susceptible MC-004 and susceptible YS-033 were excluded using the Psa filter. MC-021, MC-026, YS-031, YS-037, and YS-038 were retained as candidate elite germplasm. Heat-response class was used for characterization rather than exclusion. The collection provides regional records of taxonomic, cytotype, and phenotypic variation and identifies accessions for conservation and further evaluation. The five candidates require common-garden, multi-year, and complementary stress-response validation before their breeding value can be established.

## Figures and Tables

**Figure 1:**
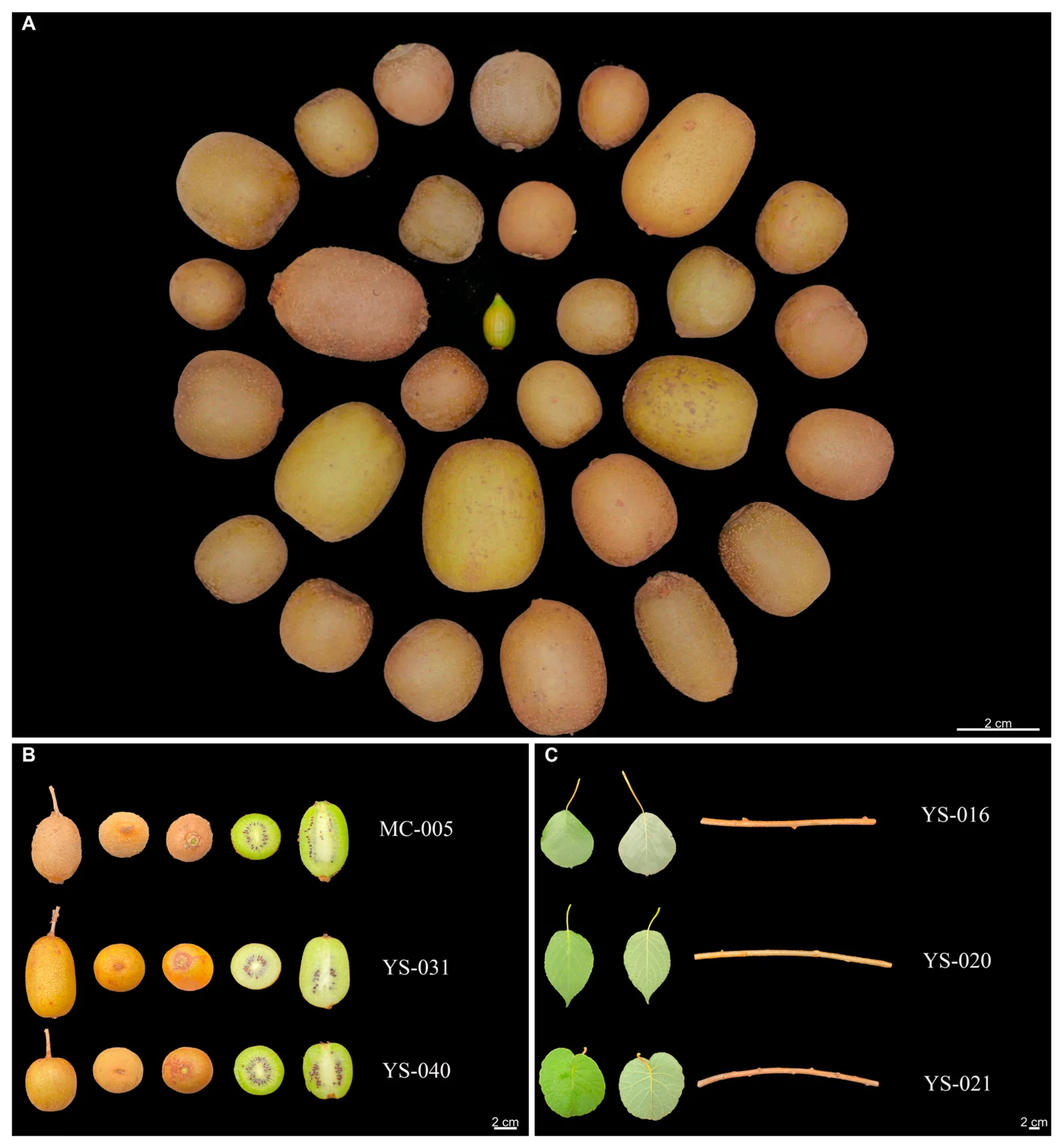
Representative morphology of wild kiwifruit accessions. (**A**) Mature fruits illustrating external morphological variation across the collection. (**B**) External views and transverse and longitudinal sections of fruits from MC-005, YS-031, and YS-040. (**C**) Paired leaf surfaces and one-year-old shoots of YS-016, YS-020, and YS-021. Scale bars = 2 cm.

**Figure 2:**
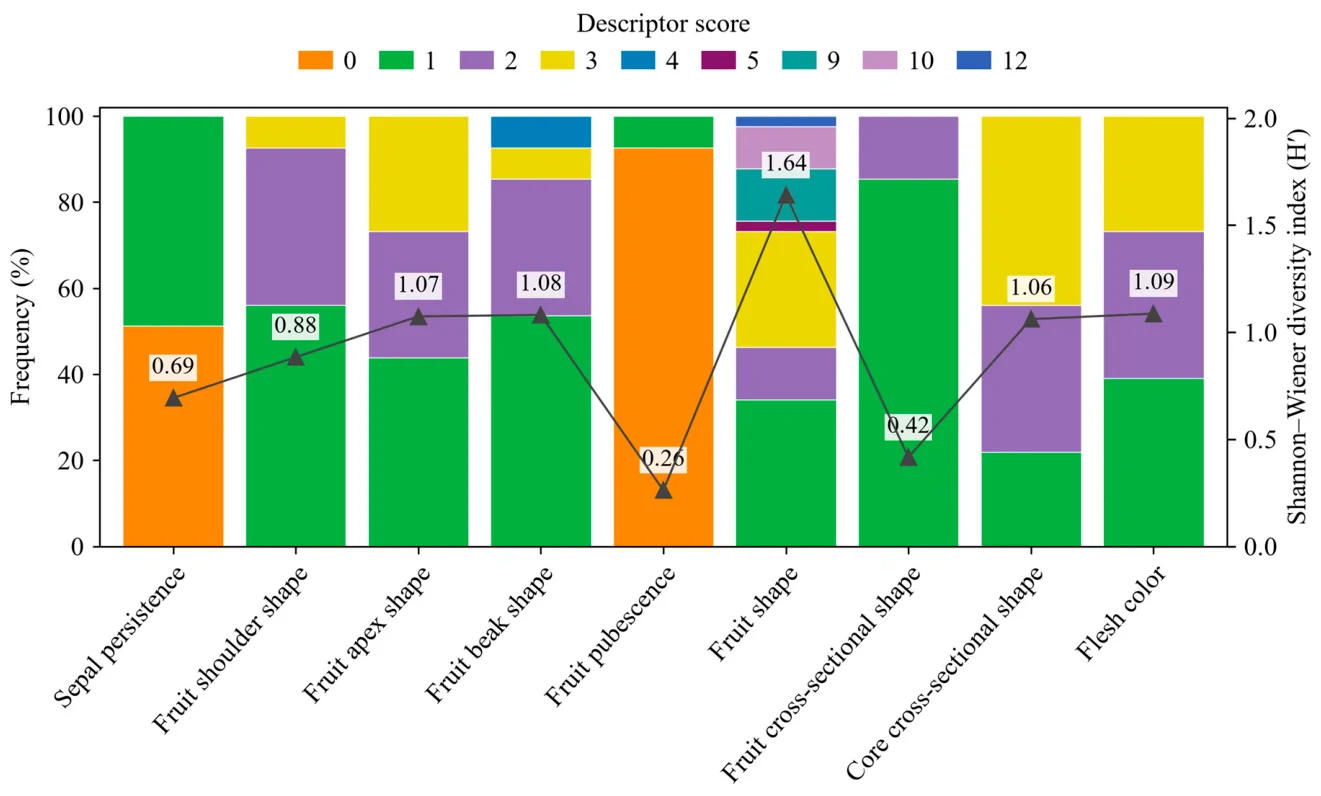
Frequency distribution and diversity of nine descriptive fruit traits in 41 wild kiwifruit accessions. Stacked bars indicate category frequencies (%), and colors represent descriptor scores. Scores follow Table S3. Sepal persistence: 0 = absent; 1 = present. Fruit shoulder shape: 1 = square; 2 = circular; 3 = sloping. Fruit apex shape: 1 = concave; 2 = flat; 3 = convex. Fruit beak shape: 1 = shallow blunt protrusion; 2 = deep blunt protrusion; 3 = shallow pointed protrusion; 4 = deep pointed protrusion. Fruit pubescence: 0 = absent; 1 = present. Fruit shape: 1 = short oval; 2 = trapezoidal; 3 = short cylindrical; 5 = globose. Additional fruit-shape scores: 9 = obovate; 10 = ellipsoid; 12 = long ellipsoid. Fruit cross-sectional shape: 1 = circular; 2 = elliptic. Core cross-sectional shape: 1 = circular; 2 = elliptic; 3 = irregular. Flesh color: 1 = green; 2 = yellow-green; 3 = yellow. The gray line and triangular markers show the Shannon–Wiener index (H′) on the right axis. Index values are shown beside the markers.

**Figure 3:**
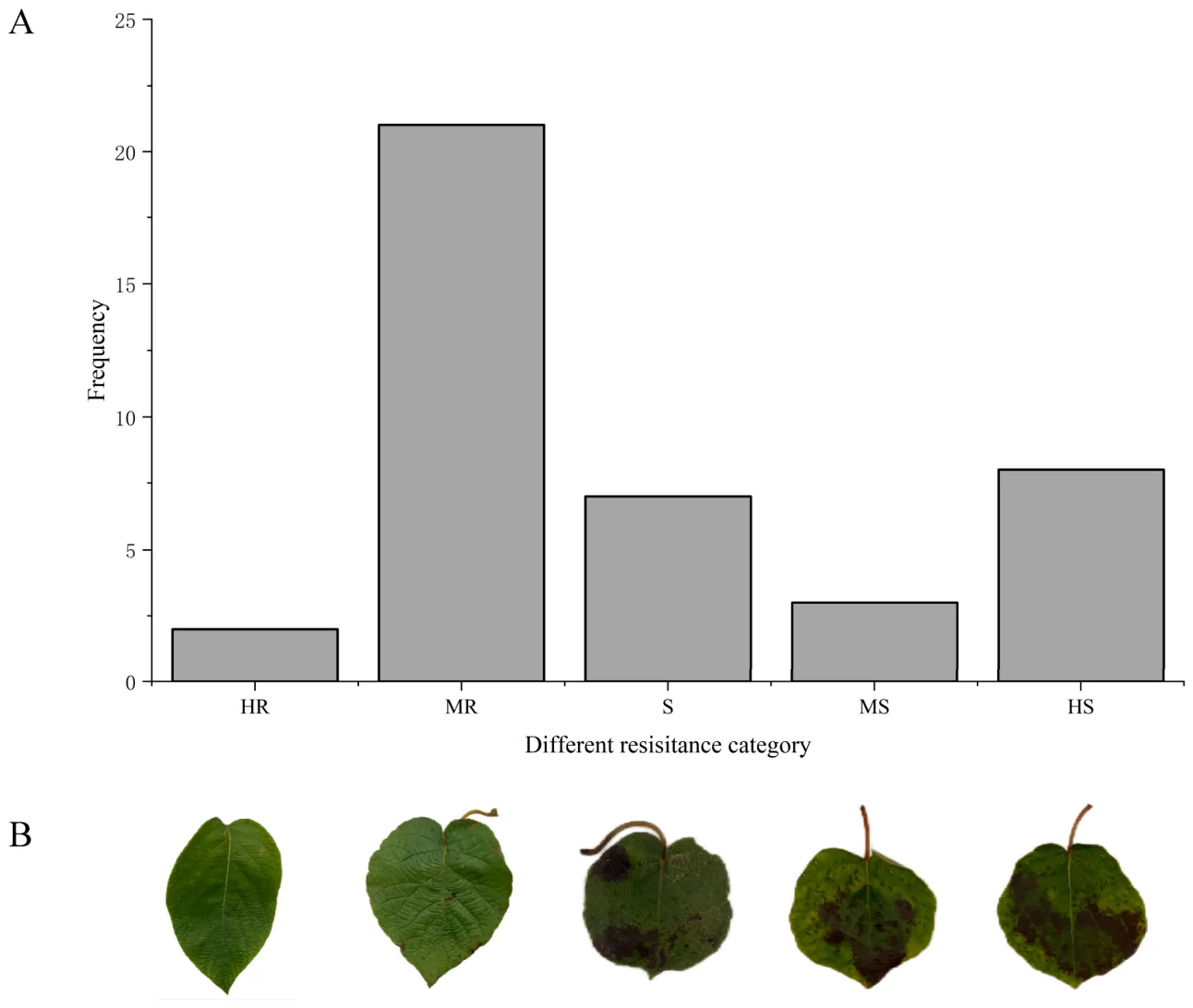
Frequency distribution of bacterial canker resistance classes (**A**) and representative lesions for each resistance class (**B**). HR, highly resistant; MR, moderately resistant; S, susceptible; MS, moderately susceptible; HS, highly susceptible.

**Figure 4:**
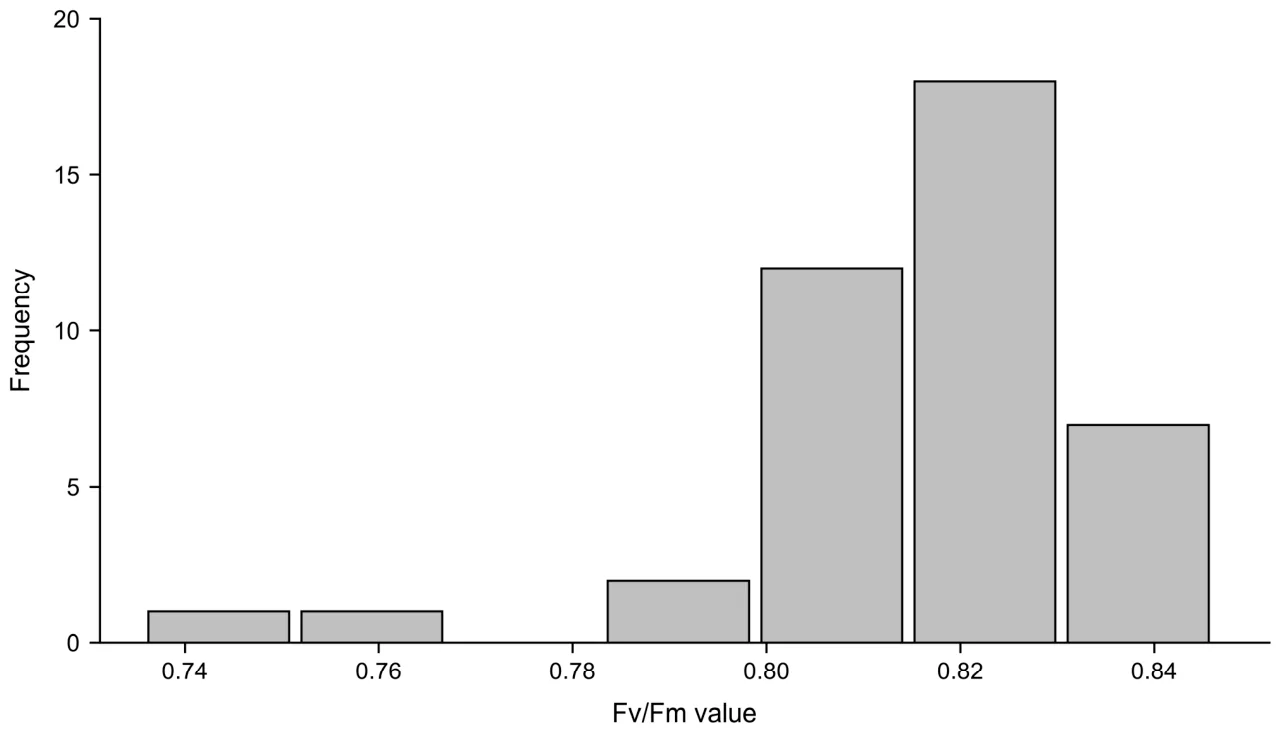
Frequency distribution of maximum quantum efficiency of photosystem II (PSII; Fv/Fm) in detached leaves.

**Figure 5:**
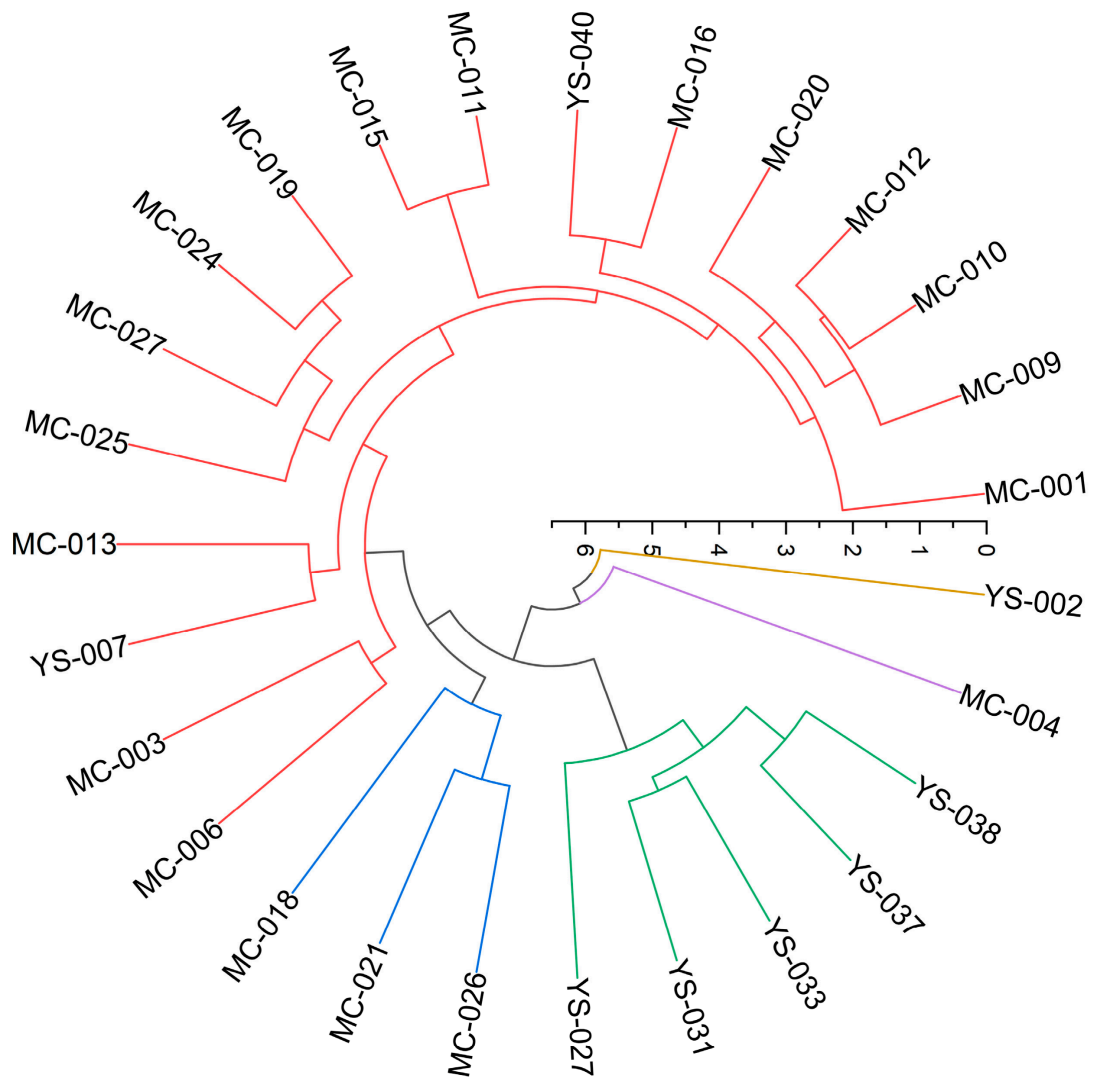
Exploratory hierarchical clustering of 27 kiwifruit accessions using nine z-standardized variables, Euclidean distance, and average linkage. Variables comprised Psa disease index, post-treatment F_v_/F_m_, dry matter content, soluble solids content, titratable acidity, vitamin C content, fruit weight, longitudinal diameter, and transverse diameter. Red, blue, green, yellow, and purple indicate Groups I, II, III, IV, and V, respectively.

**Table 1:** Distribution frequencies and diversity of fruit traits among 41 wild kiwifruit accessions.

Trait	Descriptor	Score	No. of Accessions	Percentage (%)	Shannon Diversity Index (H′)
Sepal persistence	Absent	0	21	51.22	0.69
	Present	1	20	48.78	
Fruit shoulder shape	Square	1	23	56.10	0.88
	Circular	2	15	36.59	
	Sloping	3	3	7.32	
Fruit apex shape	Concave	1	18	43.90	1.07
	Flat	2	12	29.27	
	Convex	3	11	26.83	
Fruit beak shape	Shallow blunt protrusion	1	22	53.66	1.08
	Deep blunt protrusion	2	13	31.71	
	Shallow pointed protrusion	3	3	7.32	
	Deep pointed protrusion	4	3	7.32	
Fruit pubescence	Absent	0	38	92.68	0.26
	Present	1	3	7.32	
Fruit shape	Short oval	1	14	34.15	1.64
	Trapezoidal	2	5	12.20	
	Short cylindrical	3	11	26.83	
	Globose	5	1	2.44	
	Obovate	9	5	12.20	
	Ellipsoid	10	4	9.76	
	Long ellipsoid	12	1	2.44	
Fruit cross-sectional shape	Circular	1	35	85.37	0.42
	Elliptic	2	6	14.63	
Core cross-sectional shape	Circular	1	9	21.95	1.06
	Elliptic	2	14	34.15	
	Irregular	3	18	43.90	
Flesh color	Green	1	16	39.02	1.09
	Yellow-green	2	14	34.15	
	Yellow	3	11	26.83	

Note: Each trait was assessed in 41 accessions. Percentages were calculated using this denominator, and H′ was calculated from the category proportions.

**Table 2:** Variation in major fruit traits among 27 wild kiwifruit accessions.

Statistic	Longitudinal Diameter (cm)	Transverse Diameter (cm)	Fruit Weight (g)	Soluble Solids Content (%)	Titratable Acidity (%)	Vitamin C (mg·100 g^−1^ FW)	Dry Matter Content (%)	Overall Mean
Maximum	5.85	4.46	60.01	18.87	1.62	149.77	28.86	^−^
Minimum	2.88	2.59	10.72	9.17	0.36	3.22	13.64	^−^
Mean	3.80	3.39	26.33	13.06	0.83	26.03	18.41	^−^
Standard deviation	0.77	0.44	12.45	2.71	0.28	31.34	3.24	^−^
Coefficient of variation (%)	20.17	12.92	47.29	20.74	33.67	120.41	17.61	38.97
Shannon diversity index (H′)	1.88	1.82	1.77	1.88	1.84	1.50	1.74	1.78

**Table 3:** Eigenvalues and variance contributions of the first three principal components.

Statistic	PC1	PC2	PC3
Eigenvalues	2.880	1.786	1.157
Variance explained (%)	41.136	25.513	16.523
Normalized variance contribution (%)	49.46	30.67	19.87

**Table 4:** Principal component loadings for seven fruit-quality traits in 27 wild kiwifruit accessions.

Trait	PC1	PC2	PC3
Fruit weight	0.981	0.043	0.079
Longitudinal diameter	0.939	0.114	−0.126
Transverse diameter	0.926	−0.006	0.240
Soluble solids content	0.035	0.948	−0.102
Dry matter content	−0.265	0.876	−0.037
Titratable acidity	−0.319	−0.107	0.740
Vitamin C content	0.068	0.306	0.720

**Table 5:** Principal component scores and comprehensive scores for 27 wild kiwifruit accessions.

Accession	F1	F2	F3	F	Rank	Fv/Fm	Heat Tolerance	Disease Index	Resistance
YS-038	2.27	0.35	0.20	1.27	1	0.80	MHT	28.57	MR
MC-004	1.12	−0.33	2.79	1.00	2	0.83	HT	100.00	HS
MC-021	0.44	2.20	0.05	0.90	3	0.82	HT	14.29	MR
YS-037	1.63	−0.23	0.11	0.75	4	0.82	HT	7.14	HR
MC-026	0.69	0.74	0.45	0.66	5	0.84	HT	23.81	MR
YS-033	1.26	0.65	−1.23	0.58	6	0.81	MHT	33.00	S
YS-031	1.97	−0.66	−1.25	0.52	7	0.80	MHT	21.43	MR
MC-006	−0.32	0.67	1.73	0.39	8	0.83	HT	78.57	HS
MC-018	−1.10	2.79	0.20	0.35	9	0.81	MHT	14.29	MR
YS-027	0.92	−0.70	0.23	0.29	10	0.81	MHT	78.57	HS
MC-003	−0.27	0.05	1.41	0.16	11	0.84	HT	75.00	HS
MC-024	−0.17	0.10	0.18	−0.02	12	0.83	HT	57.14	MS
MC-020	−0.23	−0.03	0.43	−0.04	13	0.81	MHT	14.29	MR
MC-019	−0.66	0.77	−0.11	−0.11	14	0.83	HT	64.29	MS
YS-040	0.34	−0.48	−0.73	−0.13	15	0.83	HT	21.43	MR
MC-025	−0.79	1.48	−1.32	−0.20	16	0.83	HT	80.95	HS
MC-027	−0.70	0.50	−0.24	−0.24	17	0.85	HT	42.86	S
MC-013	−0.02	−1.35	0.65	−0.29	18	0.81	MHT	17.86	MR
YS-002	0.47	−0.66	−1.69	−0.31	19	0.74	HS	14.29	MR
MC-009	−0.78	−0.21	−0.29	−0.51	20	0.82	HT	14.29	MR
MC-016	−0.34	−0.40	−1.11	−0.51	21	0.83	HT	50.00	S
MC-010	−0.56	−0.81	−0.17	−0.56	22	0.81	MHT	28.57	MR
YS-007	−1.04	−1.19	1.34	−0.62	23	0.84	HT	14.29	MR
MC-012	−0.91	−0.48	−0.40	−0.68	24	0.82	HT	33.33	S
MC-011	−0.81	−0.71	−0.66	−0.75	25	0.82	HT	90.48	HS
MC-015	−0.93	−1.26	−0.40	−0.93	26	0.83	HT	85.71	HS
MC-001	−1.46	−0.79	−0.15	−0.99	27	0.81	MHT	42.86	S

F = 0.4946F_1_ + 0.3067F_2_ + 0.1987F_3_. Heat tolerance: HT, heat tolerant; MHT, moderately heat tolerant; HS, heat sensitive. Resistance: HR, highly resistant; MR, moderately resistant; S, susceptible; MS, moderately susceptible; HS, highly susceptible.

## Data Availability

Data are contained within the article and Supplementary Materials.

## Supplementary Materials

The following supporting information can be downloaded at: https://www.mdpi.com/article/10.3390/plants15192894/s1, Table S1: Survey and collection of wild kiwifruit resources in the Dabie Mountains. Table S2: Chromosome ploidy of 117 wild kiwifruit accessions. Table S3: Scoring criteria for qualitative fruit traits of wild kiwifruit germplasm. Table S4: Grading criteria for resistance to kiwifruit bacterial canker. Table S5: Analysis participation of the 41 fruiting wild kiwifruit accessions.
