# Supplementary material for: Multi-Trait Characterization and Selection of Elite Wild Kiwifruit (Actinidia spp.) Germplasm from the Dabie Mountains of Hubei, China
Source: Plants (Basel). 2026 Sep 22;15(19):2894. doi: 10.3390/plants15192894 (PMC13666996; doi:10.3390/plants15192894)
Supplement: Supplementary file 1 [file plants-15-02894-s001.zip › plants-4534455-supplementary.pdf]

Supplementary Tables

Table S1. Survey and collection of wild kiwifruit resources in the Dabie Mountains.

Location	Code	Latitude (N)	Longitude (E)	Elevation (m)	No. recorded	No. of scion samples	No. of fruit samples	Species
Chenjiachong Village	MC	31°15′32″	114°47′43″	170–300	2	2	0	*A. chinensis*
Zhaojiafan Village	MC	31°27′22″	115°00′20″	180–400	27	27	15	*A. chinensis*
Xianyangyan Village	MC	31°30′21″	115°02′49″	207–268	7	7	3	*A. chinensis*; *A. chinensis var. deliciosa*
Zhujiahe Village	MC	31°27′6″	115°00′48″	300–521	18	18	8	*A. chinensis*
Daqishan Village	TF	30°52′7″	115°06′19″	265–973	20	20	0	*A. chinensis*
Huayoufan Village	LT	31°05′52″	115°40′29″	318–696	5	5	0	*A. chinensis*
Tiantangzhai Scenic Area	YS	31°05′24″	115°47′34″	790–1730	31	31	10	*A. chinensis*; *A. polygama*
Wujiashan Forest Farm	YS	31°04′30″	115°48′8″	500–730	7	7	5	*A. chinensis*
**Total**				**170–1730**	**117**	**117**	**41**	

Table S2. Chromosome ploidy of 117 wild kiwifruit accessions.

Species	No. of accessions	Ploidy		
		2x	4x	6x
A. chinensis	106	106	0	0
A. chinensis var. deliciosa	8	0	3	5
A. polygama	3	3	0	0

Table S3. Scoring criteria for qualitative fruit traits of wild kiwifruit germplasm.

Trait	Descriptor	Score
Sepal persistence	Absent	0
	Present	1
Fruit shoulder shape	Square	1
	Circular	2
	Sloping	3
Fruit apex shape	Concave	1
	Flat	2
	Convex	3
Fruit beak shape	Shallow blunt protrusion	1
	Deep blunt protrusion	2
	Shallow pointed protrusion	3
	Deep pointed protrusion	4
Fruit pubescence	Absent	0
	Present	1
Fruit shape	Short oval	1
	Trapezoidal	2
	Short cylindrical	3
	Globose	5
	Obovate	9
	Ellipsoid	10
	Long ellipsoid	12
Fruit cross-sectional shape	Circular	1
	Elliptic	2

Trait	Descriptor	Score
Core cross-sectional shape	Circular	1
	Elliptic	2
	Irregular	3
Flesh color	Green	1
	Yellow-green	2
	Yellow	3

Table S4. Grading criteria for resistance to kiwifruit bacterial canker.

Grade level	Standard grade
0	No lesions are present on the leaf.
1	Scattered lesions are present on the leaf, with a total lesion area < 1 cm².
3	A small number of lesions is present on the leaf, with a total lesion area ≥ 1 and < 2 cm².
5	A moderate number of lesions is present on the leaf, with a total lesion area ≥ 2 and < 3 cm².
7	Numerous lesions are present on the leaf, with a total lesion area ≥ 3 cm².

Table S5. Analysis participation of the 41 fruiting wild kiwifruit accessions.

Accession	Descriptive fruit traits	Psa response	Heat response	Fruit quality
MC-001	Yes	Yes	Yes	Yes
MC-002	Yes	Yes	Yes	No
MC-003	Yes	Yes	Yes	Yes
MC-004	Yes	Yes	Yes	Yes
MC-005	Yes	Yes	Yes	No
MC-006	Yes	Yes	Yes	Yes
MC-007	Yes	Yes	Yes	No
MC-008	Yes	Yes	Yes	No
MC-009	Yes	Yes	Yes	Yes
MC-010	Yes	Yes	Yes	Yes
MC-011	Yes	Yes	Yes	Yes
MC-012	Yes	Yes	Yes	Yes
MC-013	Yes	Yes	Yes	Yes
MC-014	Yes	Yes	Yes	No

Accession	Descriptive fruit traits	Psa response	Heat response	Fruit quality
MC-015	Yes	Yes	Yes	Yes
MC-016	Yes	Yes	Yes	Yes
MC-018	Yes	Yes	Yes	Yes
MC-019	Yes	Yes	Yes	Yes
MC-020	Yes	Yes	Yes	Yes
MC-021	Yes	Yes	Yes	Yes
MC-022	Yes	Yes	Yes	No
MC-023	Yes	Yes	Yes	No
MC-024	Yes	Yes	Yes	Yes
MC-025	Yes	Yes	Yes	Yes
MC-026	Yes	Yes	Yes	Yes
MC-027	Yes	Yes	Yes	Yes
YS-001	Yes	Yes	Yes	No
YS-002	Yes	Yes	Yes	Yes
YS-007	Yes	Yes	Yes	Yes
YS-012	Yes	Yes	Yes	No
YS-016	Yes	Yes	Yes	No
YS-020	Yes	Yes	Yes	No
YS-027	Yes	Yes	Yes	Yes
YS-029	Yes	Yes	Yes	No
YS-031	Yes	Yes	Yes	Yes
YS-032	Yes	Yes	Yes	No
YS-033	Yes	Yes	Yes	Yes
YS-037	Yes	Yes	Yes	Yes
YS-038	Yes	Yes	Yes	Yes
YS-039	Yes	Yes	Yes	No
YS-040	Yes	Yes	Yes	Yes

Note: The 41 accessions were fruiting during field sampling and supplied at least five fruits at a suitable developmental stage. Fourteen accessions developed soft rot or severe shriveling/dehydration during postharvest ripening and were therefore not included in fruit-quality analysis.
